# Identification of pathogens and detection of antibiotic susceptibility at single-cell resolution by Raman spectroscopy combined with machine learning

**DOI:** 10.3389/fmicb.2022.1076965

**Published:** 2023-01-04

**Authors:** Weilai Lu, Haifei Li, Haoning Qiu, Lu Wang, Jie Feng, Yu Vincent Fu

**Affiliations:** ^1^State Key Laboratory of Microbial Resources, Institute of Microbiology, Chinese Academy of Sciences, Beijing, China; ^2^College of Life Sciences, University of Chinese Academy of Sciences, Beijing, China; ^3^Savaid Medical School, University of Chinese Academy of Sciences, Beijing, China

**Keywords:** Raman spectroscopy, pathogenic bacteria, antibiotic resistance, single cell, machine learning

## Abstract

Rapid, accurate, and label-free detection of pathogenic bacteria and antibiotic resistance at single-cell resolution is a technological challenge for clinical diagnosis. Overcoming the cumbersome culture process of pathogenic bacteria and time-consuming antibiotic susceptibility assays will significantly benefit early diagnosis and optimize the use of antibiotics in clinics. Raman spectroscopy can collect molecular fingerprints of pathogenic bacteria in a label-free and culture-independent manner, which is suitable for pathogen diagnosis at single-cell resolution. Here, we report a method based on Raman spectroscopy combined with machine learning to rapidly and accurately identify pathogenic bacteria and detect antibiotic resistance at single-cell resolution. Our results show that the average accuracy of identification of 12 species of common pathogenic bacteria by the machine learning method is 90.73 ± 9.72%. Antibiotic-sensitive and antibiotic-resistant strains of *Acinetobacter baumannii* isolated from hospital patients were distinguished with 99.92 ± 0.06% accuracy using the machine learning model. Meanwhile, we found that sensitive strains had a higher nucleic acid/protein ratio and antibiotic-resistant strains possessed abundant amide II structures in proteins. This study suggests that Raman spectroscopy is a promising method for rapidly identifying pathogens and detecting their antibiotic susceptibility.

## 1. Introduction

Antimicrobial resistance (AMR) is a global public health challenge. It has been estimated that almost 5 million deaths globally are associated with bacterial AMR, including more than 1.2 million deaths attributable to AMR in 2019 (Murray et al., 2022). The number of deaths worldwide will reach 10 million a year by 2050 if the trend in rising AMR is not efficiently contained (O’neill, 2016). The emergence of AMR is ascribed to the overuse and misuse of antibiotics in clinical treatment, the livestock industry, and aquaculture (Andersson and Hughes, 2014; Aslam et al., 2018; Ben et al., 2019). Diagnostic uncertainty is a major reason for the overprescribing of antibiotics in clinical practice (Llor and Bjerrum, 2014; Michael et al., 2014; Roope et al., 2019), largely because of shortcomings of test time and accuracy in detecting infectious pathogens and antibiotic resistance (Michael et al., 2014; Dadgostar, 2019; Jamrozik and Selgelid, 2020). Methods for rapid and accurate diagnosis are highly desirable to mitigate AMR and allow rational antibiotic therapy (Germond et al., 2018; Vasala et al., 2020).

Traditional pathogenic identification involves both phenotypic and molecular methods. Generally, phenotypic diagnoses are based on bacterial growth and metabolism. For instance, bacterial identification and the antimicrobial susceptibility test (AST) are performed simultaneously by the Vitek or BD Phoenix commercial systems in clinical microbiology (Syal et al., 2017; Franco-Duarte et al., 2019). These systems evaluate bacterial growth on a series of biochemical substrates or different carbon sources to identify bacterial species. Recently, MALDI-TOF mass spectrometry has been widely used in clinical microbiology laboratories because of its high throughput and rapid performance to identify isolated bacterial colonies (Wieser et al., 2012; Strejcek et al., 2018; Vasala et al., 2020). However, these methods are laborious and require time-consuming cell culture. Moreover, many bacteria are slow growing or non-culturable in the laboratory. For example, *Mycobacterium tuberculosis* needs more than 2 weeks for cultivation, sometimes up to 6–8 weeks (Acharya et al., 2020). Molecular methods such as 16S rDNA gene sequencing and quantitative PCR provide high sensitivity and specificity, and they are also very rapid. In some cases, such as metagenomic sequencing, bacterial culture is not required for these nucleic acid-based detection methods. However, molecular methods conventionally require extensive sample preparation. Difficulties in preparing target DNA can arise when samples are present in tiny amounts or contaminated by interfering substances. Moreover, molecular methods of bacterial identification are all destructive. They cannot be utilized to identify living microbes *in situ* for real-world samples.

Raman spectroscopy is a non-invasive, culture- and label-free technique that is able to monitor the chemical composition and metabolism of single live microorganisms in real time (Lee et al., 2021). The Raman spectrum of an individual cell represents an ensemble of different molecular vibration modes and structures, including nucleic acids, proteins, lipids, and carbohydrates (Lorenz et al., 2017; He et al., 2019; Yan et al., 2021a). High dimensional and complex Raman bands provide rich information about variable cellular phenotypes to distinguish different bacteria (Germond et al., 2018; Du et al., 2020). However, it is challenging to classify different species because of the weak Raman signal of a single bacterium, spectral variations between individuals of the same species, and spectral overlaps of different molecules (Khan et al., 2018; Ho et al., 2019; Yan et al., 2021b). A variety of chemometric analyses and machine learning methods, such as principal component analysis (PCA), support vector machine (SVM), random forest (RF), and convolutional neural networks (CNN), have been employed to analyze complicated Raman spectral data (Senger and Scherr, 2020; Xu et al., 2020). Of these, machine learning has shown great promise in rapidly and accurately identifying microorganisms at single-cell resolution (Ho et al., 2019; Lu et al., 2020; Zhou et al., 2022). Besides Raman-based identification of bacteria, Raman spectroscopy has also been used to determine bacterial antibiotic resistance when coupled with stable isotope probing, such as heavy water labeling (Yang et al., 2019; Zhang et al., 2020; Yi et al., 2021). However, this method requires pre-labeling and culturing bacteria in the presence of antibiotics. In theory, the label-free signatures of bacterial Raman spectra are excellent phenotypic indicators of antibiotic resistance (Germond et al., 2018; Verma et al., 2021). It is worth exploring the possibility of identifying bacterial species and detecting antibiotic susceptibility simultaneously using single-cell Raman spectra.

In this study, we developed a method combining Raman spectroscopy with machine learning to identify a pathogenic bacterium and predict its antibiotic resistance rapidly and accurately at single-cell resolution. The average accuracy of identification of 12 species of common pathogenic bacteria by the machine learning model was 90.73 ± 9.72%. The optimal machine learning model predicted the antibiotic susceptibility of *Acinetobacter baumannii* isolated from hospital patients with 99.92% accuracy. Meanwhile, we found that antibiotic-resistant *A. baumannii* strains showed more abundant amide II structures in proteins and a lower nucleic acid/protein ratio than antibiotic-sensitive strains.

## 2. Materials and methods

### 2.1. Bacterial and yeast strains

The pathogens used in this study included seven species of gram-negative bacteria (*A. baumannii*, *Enterobacter cloacae*, *Escherichia coli*, *Klebsiella pneumonia*, *Pseudomonas aeruginosa*, *Salmonella enterica*, and *Vibrio parahaemolyticus*), three species of gram-positive bacteria (*Staphylococcus aureus*, *S. epidermidis*, and *Streptococcus pneumoniae*), and two species of fungi (*Cryptococcus neoformans* and *Candida albicans*). Five clinical strains of *A. baumannii* (ST2 sequence type) were isolated from sputum of different patients in the intensive care unit of Cangzhou Central Hospital, Hebei Province, China. The five clinical *A. baumannii* strains all contained the carbapenem resistance gene *oxa23*, which was confirmed by PCR assays as previously described (Woodford et al., 2006; Jinshu et al., 2022).

### 2.2. Single-cell Raman spectral measurements

The bacterial and yeast cells were cultured in Luria–Bertani (LB) medium or yeast extract peptone dextrose (YPD) medium with various culture times under different culturing conditions. The microbial cells were collected and suspended in 0.85% NaCl solution. A total of 20 μl suspended cells was injected in the sealed chamber for Raman spectral measurement. The Raman spectra were measured by laser tweezers Raman spectroscopy (LTRS) as previously described (Lu et al., 2020). Raman spectral calibration of the LTRS was performed at 620.9 cm^−1^, 1001.4 cm^−1^, and 1602.3 cm^−1^ of the 10 μm polystyrene spheres. The integration time was set at 60–90 s. For each species, the Raman spectra of at least 300 single cells collected from different batches were acquired. These Raman spectra were training data used for training the machine learning models. To test the classifying accuracy of the machine learning models, 80 single cells per species were gathered from other culturing batches, which were completely different from the batches used for model training. The Raman spectra acquired from these single cells were testing data, used for testing the models.

### 2.3. Raman spectral data processing and training of machine learning models

The spectra were pre-processed as follows: Savitzky–Golay smoothing to remove noise and polynomial fitting to remove the fluorescence background, followed by min–max normalization. The spectral range between 555 cm^−1^ and 1815 cm^−1^ was selected for model training and model testing. We used algorithms based on random forest (setting, 101 decision trees), support vector machine (selecting linear kernel), decision tree (using C5.0 algorithm), bagging (loading parallel backend), and naive Bayes (no Laplace correction) to build models for training Raman spectral data. A 10-fold cross-validation of the training dataset was used to evaluate the robustness of the models. In this study, the training time for the five models was about 4 hours using a HP workstation (16G, i7-8550U CPU). Independent spectra acquired from 80 single cells of each species were used to assess the performance of models. Sample identification included the processes of sample preparation (cell collection for 1–2 min), Raman spectral acquisition (integration time for 60–90 s), and species prediction (less than 30 s).

### 2.4. The architecture of the antibiotic susceptibility detection model and training details

The antibiotic susceptibilities of all six *A. baumannii* strains (one reference strain and five clinical isolates) were first determined by the VITEK 2 instrument (bioMérieux, France) according to the instruction manual. The antibiotics used in the test included imipenem, meropenem, ampicillin, cefoperazone, and cefepime. The reference strain and all the clinical isolates were cultured in LB medium without adding any antibiotics, and then the cells were harvested to acquire the Raman spectra. Data on the antibiotic resistance of each strain are listed in Supplementary Table S1. The training data for constructing the RF antibiotic susceptibility detection model included 523 spectra acquired from different *A. baumannii* strains. An independent testing dataset of 1,255 spectra from six *A. baumannii* strains was collected for testing the predictive performance of the identification models and the antibiotic susceptibility detection model. The RF antibiotic susceptibility detection model with 101 decision trees was evaluated by 10-fold cross-validation. Subsequently, the model with the highest prediction accuracy was selected. The receiver operating characteristic (ROC) curve was used to assess the diagnostic accuracy of the antibiotic susceptibility detection model in terms of antibiotic resistance. The *A. baumannii* strains were grouped based on the antimicrobial susceptibility test (Supplementary Table S1). The Raman spectra of strain ZB180325 were labeled as sensitive to imipenem and cefoperazone. The Raman spectra of strain ZB180589 were labeled as sensitive to cefoperazone and ampicillin. The Raman spectra of strains ZB180791 and ZB18102 were labeled as sensitive to imipenem and meropenem, respectively. We trained the RF model on the four-antibiotic prediction task. Completely different batches of the Raman spectra of strains were used to test the model. The main features of the Raman spectra of antibiotic-resistant and antibiotic-sensitive *A. baumannii* were analyzed by principal component analysis (PCA).

The Raman spectral pre-processing, building machine learning models, PCA analysis, and statistical analysis were all performed using the R language (R ≥ 3.6.1). In this study, the main open-source packages of R included “caret” and “hyperSpec.”

## 3. Results

### 3.1. Raman spectra of 12 common clinical pathogens

**Figure 1:**
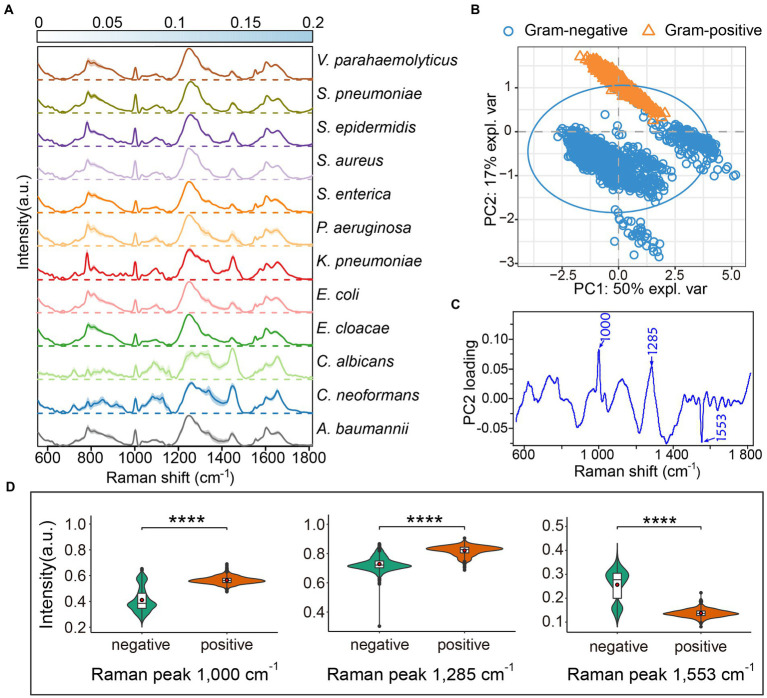
Average Raman spectra of 12 common clinical pathogens. **(A)** The average Raman spectra of each species are shown by the solid line, and the standard deviations are represented by the shadow. The standard deviations for each pathogen are presented by heatmaps to indicate the variations of Raman bands. For each species, the Raman spectra of more than 300 single cells were acquired by LTRS. **(B)** Principal component analysis (PCA) between gram-positive and gram-negative bacteria. The explained variance (expl. var) of the first two PCs are 50 and 17%, respectively. The confidence ellipse of each strain is 0.95. **(C)** The loading of PC2 in PCA analysis. **(D)** Raman intensity of gram-positive and gram-negative bacteria at peaks 1,000, 1,285 and 1,553 cm^−1^, shown by violin plots. Embedded box plots represent the median and first and third quartiles, with the whiskers representing the minimum and maximum values within 1.5 interquartile ranges from the first and third quartiles. Red dots are average Raman intensity. Two sided *t*-tests were applied to compare the statistical significances between gram-positive and gram-negative bacteria. ****p ≤ 0.0001.

To gather a training dataset, we measured the Raman spectra of 12 different pathogens, including seven gram-negative bacteria (*A. baumannii*, *Enterobacter cloacae*, *Escherichia coli*, *Klebsiella pneumonia*, *Pseudomonas aeruginosa*, *Salmonella enterica*, and *Vibrio parahaemolyticus*), three gram-positive bacteria (*Staphylococcus aureus*, *S. epidermidis*, and *Streptococcus pneumoniae*), and two fungi (*Cryptococcus neoformans* and *Candida albicans*). To minimalize batch effects on the final classification, we constructed a reference dataset of 3,982 spectra from bacteria and yeast under different culturing conditions to cover a more varied cellular physiological status and heterogeneity in the same species. The normalized average Raman spectra of the 12 pathogens with the standard deviations (SD) are shown in Figure 1A. The Raman spectral profiles of bacteria and fungi show a big visual difference. Although the Raman spectra of the 10 pathogenic bacteria look similar in pattern, the changes of Raman intensities and the SD among the different pathogens show obvious differences at the same Raman shift (Figure 1A). Subsequently, we analyzed the seven species of gram-negative bacteria and three species of gram-positive bacteria by principal component analysis (PCA). The PCA plot suggests that the gram-positive and gram-negative bacteria are separated in two clusters (Figure 1B). The most common differences between Raman peaks of the two categories were at 1000, 1285, and 1,553 cm^−1^ (Figure 1C). It is likely that the gram-positive bacteria have higher levels of phenylalanine (1,000 cm^−1^) and protein (1,285 cm^−1^; Choi et al., 2018). The gram-negative bacteria possess abundant amide II structures in proteins (Figure 1D). The spectral differences indicate that the composition and concentration of biomolecules are different for different species, and so it will be possible to extract the characteristics of different pathogens based on these informative variables.

### 3.2. Machine learning for pathogen identification from Raman spectra

As the 10 pathogenic bacteria bear a high resemblance in their Raman spectra, it is challenging to discern the subtle spectral difference between pathogens. Machine learning is a computer-based strategy that can extract subtle variation of sophisticated hidden features within Raman spectra. Here, we compared the identification accuracy of five machine learning methods: random forest (RF), support vector machine (SVM), naive Bayes (NB), bagging, and decision tree (DT). The Raman dataset of 3,982 spectra was used to train the machine learning models. We used 10-fold cross-validation to evaluate the discriminative ability of these models. In this process, one fold was randomly split out and used to validate the model trained by all the other nine folds. This process was repeated until each of the 10 folds had acted as the test set once. Taking into account the identification accuracy and successes occurring by chance, two metrics of accuracy and Cohen’s kappa (Cohen, 1960; García et al., 2009; Vieira et al., 2010) were utilized to evaluate the robustness of these models. The accuracies of both RF and SVM were higher than DT, bagging, and NB (Supplementary Figure S1), indicating that the performances of the RF and SVM models were superior to the other models for accurately identifying pathogens at the single-cell resolution. Likewise, the kappa scores for both RF and SVM were higher than the other three models. The data demonstrate that these two models have good consensus agreement for classifying microbial pathogens.

**Figure 2:**
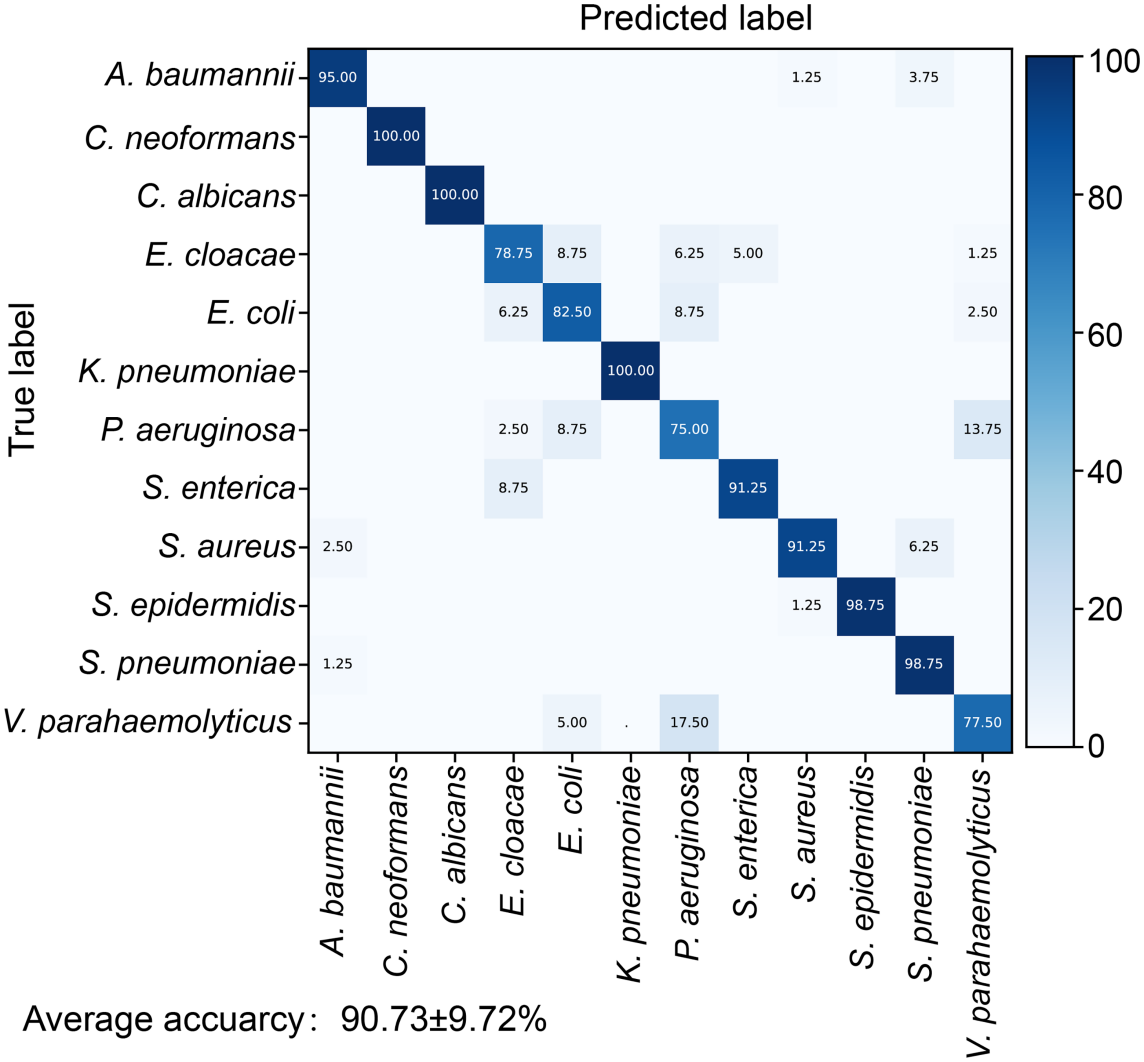
Identification accuracy of the random forest identification model for 12 pathogens. The confusion matrix shows the percentage of accurate prediction for each pathogen.

We further tested the models on the independent test dataset gathered from a separately cultured batch that consisted of 960 spectra for the 12 pathogens (80 spectra per pathogen). Four indicators including accuracy, kappa, recall, and F1 score were selected to evaluate the performances of the different machine learning models. The results suggested that the RF is slightly superior to SVM (Supplementary Table S2). The RF model identified the 12 pathogens with an average accuracy of 90.73 ± 9.72% (Figure 2). For the two fungal pathogens, *C. albicans* and *C. neoformans*, the accuracy of fungi identification reached 100%. The identification accuracies for the gram-positive bacteria *S. epidermidis* and *S. pneumonia* were 98.75%, higher than for *S. aureus* with an accuracy of 91.25%. The classifying accuracy for *K. pneumoniae* was 100%, the highest accuracy of the gram-negative bacteria. *A. baumannii* was the second highest accuracy, at 95%. The identifying accuracy for *E. coli* was 82.5%. The accuracies for the other three gram-negative bacteria (*E. cloacae*, *P. aeruginosa*, and *V. parahaemolyticus*) were between 75 and 83%.

### 3.3. Detection of the *Acinetobacter baumannii* antibiotic-resistant strain by machine learning

Species-level identification is only the first step in clinical practice, choosing the correct antibiotic against bacterial infections is more important for clinical outcome. To step toward a culture-free antibiotic susceptibility test using Raman spectroscopy, we used five multidrug resistant (MDR) *A. baumannii* strains isolated from patients in the intensive care unit (ICU; Supplementary Table S1) as a proof-of-concept. *A. baumannii* is a ubiquitous opportunistic pathogen that is responsible for a broad range of severe nosocomial infections such as bloodstream infections (Antunes et al., 2014), especially in the ICU and immunocompromised patients (Eliopoulos et al., 2008; Vázquez-López et al., 2020). Carbapenem-resistant *A. baumannii* has been listed at the top of the greatest threat list by the World Health Organization (WHO; Abadi et al., 2019; Vázquez-López et al., 2020; Murray et al., 2022). Rapid and accurate diagnosis of antibiotic resistance is critical to allow timely performance of an effective therapeutic scheme (Butler et al., 2019).

**Figure 3:**
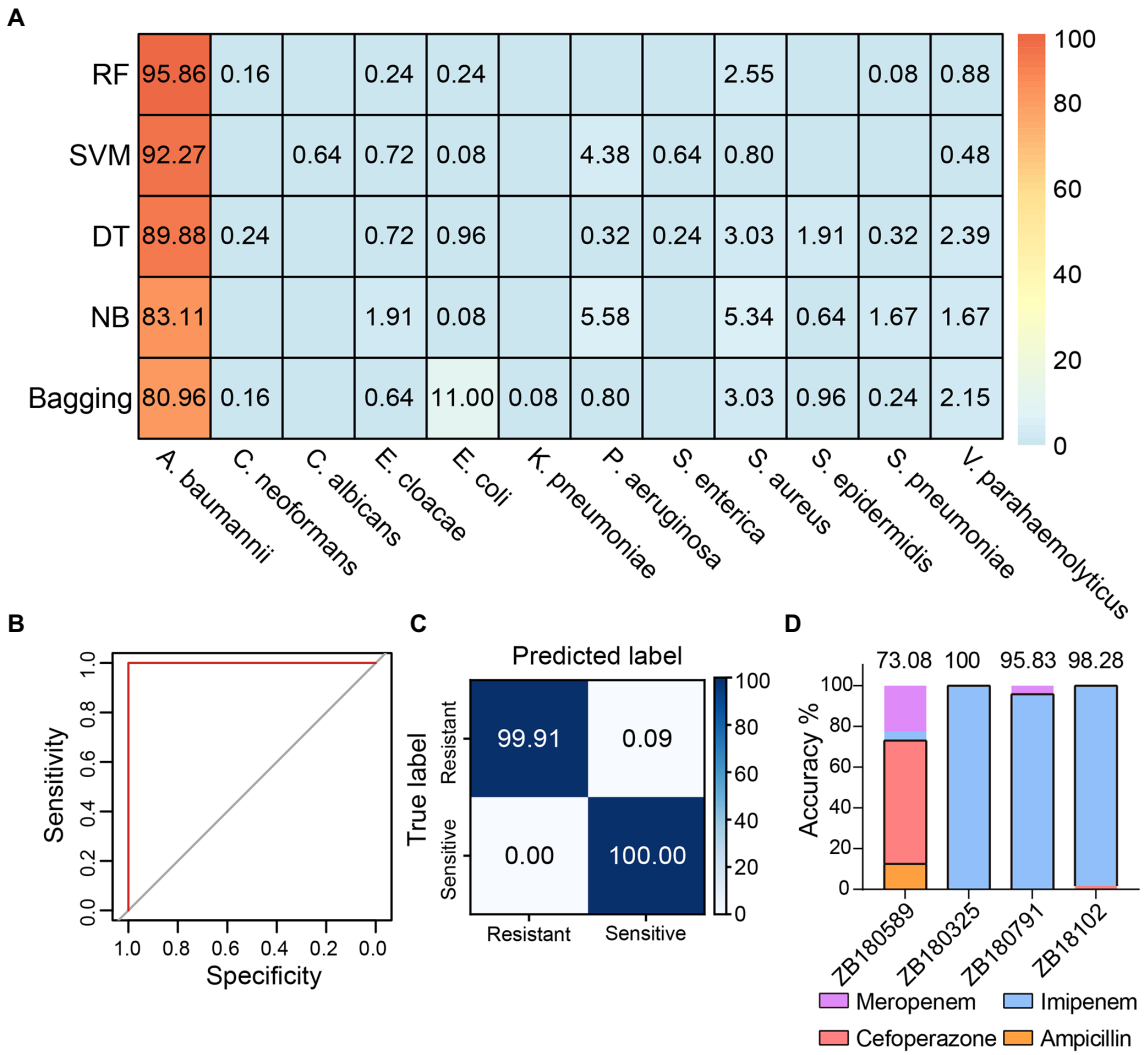
Detection of multidrug-resistant *Acinetobacter baumannii*. **(A)** The identification accuracies achieved by different models using the 1,255 Raman spectra acquired from different *A. baumannii* strains. RF: random forest, SVM: support vector machine, DT: decision tree, NB: naive Bayes. **(B)** The receiver operating characteristic (ROC) curve of discriminating antibiotic-resistant bacteria by RF model based on the Raman spectra. Both the average AUC values of resistant and sensitive bacteria are 1. **(C)** The confusion matrix shows that the RF antibiotic susceptibility detection model distinguishes between antibiotic-resistant and -sensitive *A. baumannii* strains with 99.92 ± 0.06% accuracy. **(D)** The antibiotic predictions for treatment of *A. baumannii* by the RF model. The black boxes indicate the proportion correctly predicted.

We cultured one antibiotic-sensitive *A*. *baumannii* strain (isolate code: ZB18051) and five multidrug-resistant strains (isolate codes: ZB18101, ZB18102, ZB180325, ZB180589, and ZB180791) to acquire single-cell Raman spectra. To avoid the effects of residual antibiotics from cell culture when discerning the antibiotic-resistant strain, the multidrug-resistant strains and the drug-sensitive strain were cultured in the same LB medium without supplementing antibiotics. The 523 Raman spectra from the antibiotic-sensitive strain ZB18051 and four randomly selected multidrug-resistant strains (isolate codes: ZB18101, ZB18102, ZB180325, and ZB180589) were collected to build a training dataset. The training dataset was used to train the antibiotic susceptibility detection model. A separate testing dataset, consisting of 1,255 spectra from six *A. baumannii* strains, was prepared for the model validation. First, we used the pathogenic identification models constructed previously to predict the 1,255 spectra of *A. baumannii*. The RF identification model achieved the highest identification accuracy of 95.86%, which is consistent with the previous prediction on 80 *A. baumannii* spectra (Figure 3A). This further confirmed that RF is the best identification model.

Then, an RF model was constructed to distinguish antibiotic resistance from sensitivity using the training dataset. The sensitivity (true positive rate) and specificity (true negative rate) were evaluated by the receiver operating characteristic (ROC) curve (Figure 3B). The value of AUC (the area under the ROC curve) was 1, suggesting that the RF model detects antibiotic-resistant and -sensitive *A. baumannii* with very high specificity and sensitivity based on the Raman spectra. As seen in Figure 3C, for the antibiotic-sensitive strains, the rate of correct detection was 100%. For the antibiotic-resistant strains, just 0.09% of cells were mistakenly predicted as sensitive cells. Thus, the mean accuracy predicted by the RF model reached 99.92 ± 0.06% (Figure 3C). These results indicate that Raman spectroscopy combined with RF is not only a reliable approach to accurately identify pathogens at single-cell resolution, but is also able to accurately distinguish between antibiotic-resistant and antibiotic-sensitive bacteria without labeling and antibiotic treatment. It is desirable in clinical practice to choose the correct antibiotics to treat infectious diseases. Four antibiotics (imipenem, meropenem, cefoperazone, and ampicillin) are available to treat *A. baumannii* strains (Supplementary Table S1). The *A. baumannii* strains were grouped based on the antimicrobial susceptibility test of *A. baumannii*. The independent training and testing data of *A. baumannii* were used to train and test the RF model, respectively. The average accuracy predicted by the RF model was 91.80%. The RF model accurately predicted the preferential antibiotics for the treatment of multidrug-resistant *A. baumannii* (Figure 3D). This result was also consistent with the results of testing with the VITEK 2 system (Supplementary Table S1). This implies that Raman spectroscopy combined with machine learning is able to identify the species of bacteria *in situ*, detect the antibiotic susceptibility, and facilitate the correct antibiotic choice at single-cell resolution.

### 3.4. Antibiotic resistance characteristics of *Acinetobacter baumannii*

**Figure 4:**
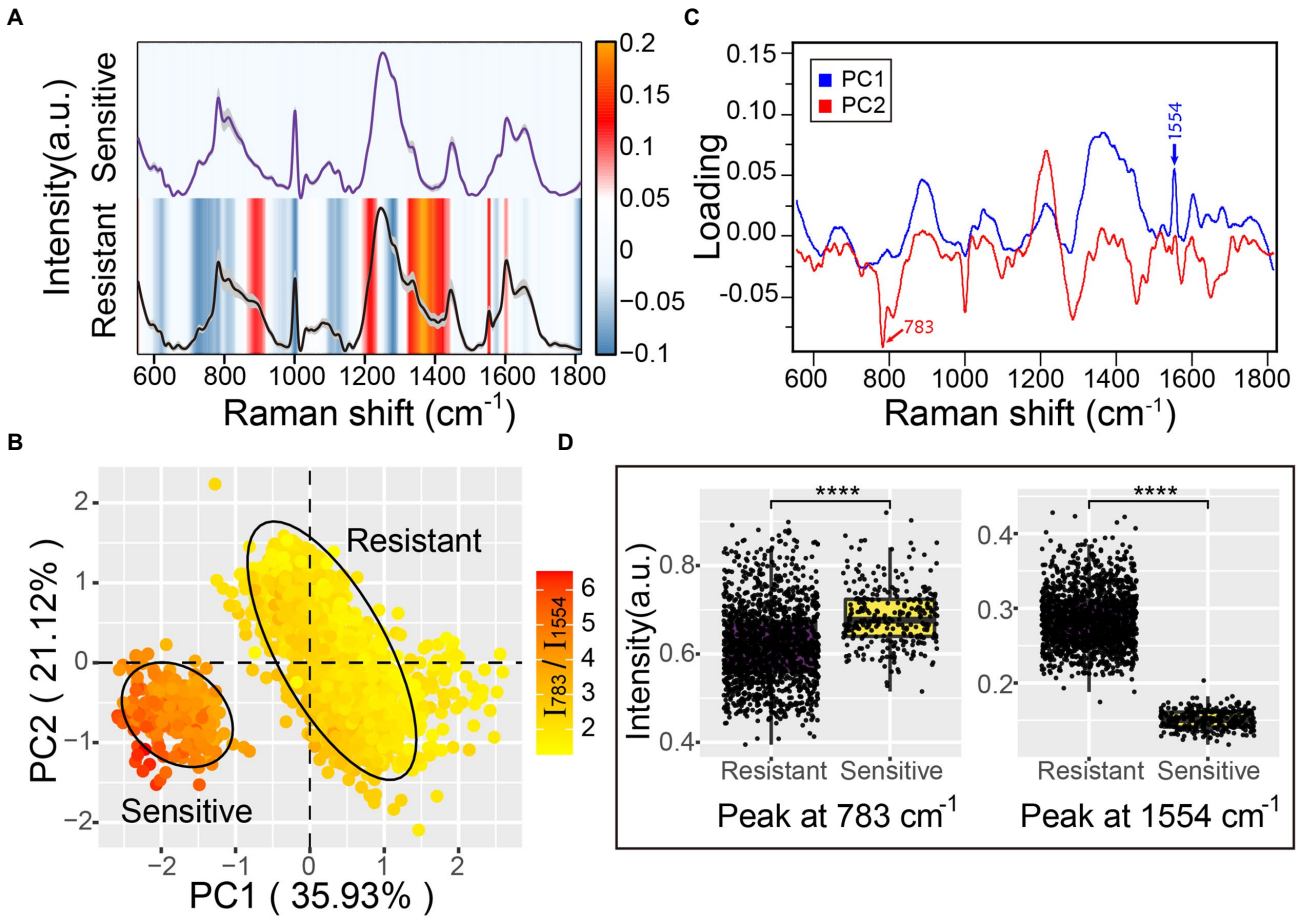
Antibiotic resistance characteristics of *A. baumannii* single-cell Raman spectra. **(A)** Raman spectra of antibiotic-sensitive and antibiotic-resistant *A. baumannii*. The average Raman spectra are shown by a solid line and the standard deviations are represented by the gray shadow. The heatmaps indicate the spectral difference between antibiotic-resistant and antibiotic-sensitive bacteria. **(B)** Principal component analysis between antibiotic-resistant and antibiotic-sensitive strains. The explained variance of the first two PCs are 35.93 and 21.12%, respectively. The confidence ellipse of each strain is 0.95. The color gradient represents the intensity ratio of the Raman peaks at 783 cm^−1^ and 1,554 cm^−1^ (I_783_/I_1554_). **(C)** The loading of PC1 and PC2 in PCA analysis. **(D)** Raman intensity of antibiotic-resistant and antibiotic-sensitive strains at the 783 cm^−1^ and 1,554 cm^−1^ peaks. Box plots represent the median and first and third quartiles, with the whiskers representing the minimum and maximum values within 1.5 interquartile ranges from the first and third quartiles. Two-sided *t*-tests were applied to compare the statistical significance between antibiotic-resistant and antibiotic-sensitive strains. ****p ≤ 0.0001. Each dot represents the Raman intensity of a single *A. baumannii* cell.

Raman spectroscopy offers rich information on chemical compositions such as DNA/RNA, proteins, lipids, and carbohydrates, which are linked with cellular physiological functions, biochemical metabolism, and transcriptomic features (Germond et al., 2018; He et al., 2021; Cui et al., 2022). Since Raman spectral signatures of multidrug-resistant *A. baumannii* contribute to the discrimination of antibiotic-resistance phenotypes, we sought to detect the corresponding physiological or metabolic features of bacterial resistance based on the Raman spectra. The Raman spectrum difference was calculated by subtracting the average spectrum of antibiotic-sensitive *A. baumannii* from the average spectrum of antibiotic-resistant *A. baumannii*. The Raman bands at 860–918 cm^−1^ (polysaccharides and proteins), 1,336–1,367 cm^−1^ (carbohydrates and proteins), 1,554 cm^−1^ (amide II of proteins), and 1,602 cm^−1^ (C–C or C–N protein bonds) were increased in the antibiotic-resistant strains (Figure 4A; Supplementary Figure S2; Table 1). In contrast, Raman peaks at 729, 783, and 1,576 cm^−1^ (DNA/RNA Raman bands) were decreased in the antibiotic-resistant strains. In addition, the Raman peaks at 1,002 cm^−1^ (phenylalanine) and 1,281 cm^−1^ (amide III of protein) in antibiotic-sensitive *A. baumannii* were also slightly increased (Figure 4A; Supplementary Figure S2; Table 1). The Raman band changes reflect the differences between antibiotic-resistant and antibiotic-sensitive strains in the composition and proportion of carbohydrates, proteins, and nucleic acids.

**Table 1:** Assignment of Raman bands in difference spectra between antibiotic-resistant and antibiotic-sensitive *Acinetobacter baumannii*.

Strains	Wavenumber (cm^−1^)	Assignment/ wavenumber (cm^−1^)	Reference
Resistant	860–918	Polysaccharides (868), hydroxyproline, tryptophan (879–880), protein (890), saccharide (891), glucose (913)	Hanlon et al., 2000; Krafft et al., 2005; Movasaghi et al., 2007; Kendall et al., 2011
	1,330–1,367	CH_2_/CH_3_, twisting, collagen (1336), tryptophan (1359), glucose (1343), protein (1270–1,335)	Cheng et al., 2005; Krafft et al., 2005; Movasaghi et al., 2007; Ramirez-Mora et al., 2019
	1,445	CH_2_ /CH_3_ of carbonic acid, cholesterol (1440–1,460), phospholipids (1445)	Maquelin et al., 2002; Töpfer et al., 2019; Park et al., 2021
	1,554	Amide II of protein	Faoláin et al., 2005; Movasaghi et al., 2007
	1,602	C–C, C–N of protein (1061)	Maquelin et al., 2002; Kuhar et al., 2021
Sensitive	729	Adenine of DNA/RNA(728)	Neugebauer et al., 2010
	783	Cytosine, uracil ring of DNA/RNA	Maquelin et al., 2002; Movasaghi et al., 2007; Kendall et al., 2011
	811	O–P–O of lipids	Notingher et al., 2003
	1,002	Phenylalanine of protein	Töpfer et al., 2019
	1,099	CC skeletal and C–O–C glycosidic link (1098)	Maquelin et al., 2002
	1,125	C–C skeletal of acyl backbone in lipid (1126)	Cheng et al., 2005
	1,281	Amide III of protein CH_2_ wagging vibrations from glycine backbone (1280)	Dukor, 2001
	1,576	Guanine, adenine of DNA/RNA (ring stretching) (1575)	Maquelin et al., 2002

To further reveal the primary differences between antibiotic-resistant and antibiotic-sensitive *A. baumannii*, principal component analysis (PCA) was used to reduce the dimensionality of the spectra data and extract the main features. The first two principal components (PC1 and PC2) projected by the Raman spectral data accounted for 57.05% of the original variance. The scores plot of PC1 and PC2 suggested that the antibiotic-resistant and antibiotic-sensitive *A. baumannii* were completely separated in two clusters (Figure 4B). The two clusters represented a significant difference in Raman spectra along the PC1 score values. The Raman peak at 1,554 cm^−1^ showed a clear positive correlation to resistant bacteria. The loading of PC1 and PC2 indicated that the Raman spectral difference between antibiotic-resistant and antibiotic-sensitive strains was predominantly assignable to the proteins and nucleic acids signals (Figure 4C). The PC1 loadings confirmed that the amide II Raman intensity at 1,554 cm^−1^ was increased for the antibiotic-resistant *A. baumannii* (Figures 4A,D; Supplementary Figures S2, S3). The data indicate that antibiotic-resistant strains possess abundant amide II structures in proteins. This might reflect active synthesis of oxacillinase enzyme in the *A. baumannii* strain containing the *oxa23* gene, even in the absence of antibiotic in the environment. In contrast to antibiotic-resistant *A. baumannii*, the Raman signal of nucleic acids in the antibiotic-sensitive strain showed a higher intensity at the 783 cm^−1^ peak (Figures 4A,D; Supplementary Figures S2, S3). Moreover, the average ratio of the Raman intensity for I_783_/I_1554_ (nucleic acids/proteins) was 2.20 in the antibiotic-sensitive strain, while the average ratio of I_783_/I_1554_ in the antibiotic-resistant strains was close to 4.58 (Figure 4B).

## 4. Discussion

Rapid pathogen identification and diagnosis of antibiotic susceptibility are critical for efficient clinical therapy and deceleration of AMR emergence. In this study, we applied machine learning techniques to Raman spectroscopy to identify 12 common clinical pathogens at single-cell resolution. We also showed that the machine learning model can detect single antibiotic-resistant *A. baumannii* cells with high accuracy based on the Raman spectrum. Significantly, the combination of Raman spectra and machine learning could predict the bacterial antibiotic resistance in the absence of antibiotic treatment. We envision that it could be important to develop a method to detect the drug resistance of pathogens *in situ*. Such an approach combined with an automated system would rapidly and accurately identify each microbial cell in clinical samples, provide an opportunity for analyzing the biochemical and metabolic characteristics of each cell, and could even directly explore pathogenic ecophysiology in native habitats.

This study uses five machine learning algorithms (RF, SVM, DT, NB, and bagging) to construct prediction models. Compared with the accuracy of the other models, the SVM and RF models had the best performance. The average accuracy of the RF identification model to identify 12 common clinical pathogens was 90.73%, and the RF antibiotic susceptibility detection model predicted the antibiotic susceptibility of *A*. *baumannii* with an accuracy of 99.92%. Thus, RF is the best machine learning algorithm for construction of a prediction model in this study. RF works by various independent decision trees that vote on the pathogens and output the category labels for those have the majority vote (Biau and Scornet, 2016; Shaikhina et al., 2019). This method might be more robust than the single DT and bagging methods (Cui et al., 2020). Moreover, RF model training takes less time than for DT and SVM (Parmar et al., 2019), which is a merit when applying this to larger datasets.

Antibiotic resistance is a consequence of the immense genetic plasticity of bacterial pathogens. Understanding the molecular mechanisms of resistance is of paramount importance to design strategies for curtailing the emergence and spread of resistance. This study lays the foundation to infer the mechanism of antibiotic resistance from Raman spectral signatures. Compared with the antibiotic-sensitive *A. baumannii* strain, the Raman spectral differences of five antibiotic-resistant strains were almost consistent, especially at the Raman peaks of proteins (1,554 and 1,602 cm^−1^) that show significant increased intensity. Since all five strains contain the *oxa23* gene that encodes the oxacillinase enzyme, the stronger protein Raman peaks might be related to the high expression level of oxacillinase *in vivo*. In addition, the increased phospholipids (1,445 cm^−1^), polysaccharides and proteins (860–918 cm^−1^ and 1,330–1,367 cm^−1^) in antibiotic-resistant *A. baumannii* might promote changes in the bacterial cell membrane, resulting in enhanced biofilm formation (Ramirez-Mora et al., 2019; Gieroba et al., 2020; Park et al., 2021). Because of the complex and high-dimensional nature of the spectra, representing many biomolecules, it remains a challenge to assign a Raman spectrum wavenumber directly to a specific biomolecule to infer the molecular mechanism of bacterial antibiotic resistance. More efforts are required to develop computational methods to allow mapping between Raman spectra and biomolecular profiles. Meanwhile, the integration of big data obtained from single-cell genomes, transcriptomes, proteomes, and metabolomes with machine learning would assist in revealing the bacterial drug-resistance mechanism based on the Raman spectra.

The antibiotic-resistant *A. baumannii* strains used in this study have multiple drug resistance, including imipenem, ampicillin, sulfamethoxazole, ceftriaxone, and levofloxacin (Supplementary Table S1). This multiple drug resistance makes clinical anti-infective treatment more difficult and confers one of the most serious threats to public health. The RF antibiotic susceptibility detection model built in this study can only distinguish resistant strains from sensitive strains. This model is, thus far, unable to discern the specific antibiotic resistance. Future study will focus on applying deep learning in modeling to determine the spectral characteristics of resistance to each specific antibiotic. Such a technique would allow for accurate treatment and would limit MDR.

Although Raman spectroscopy has not yet been applied to pathogenic identification and antibiotic susceptibility detection in clinical practice, a standardized Raman spectral database of pathogenic microorganisms covering more cell physiological states, growth media and conditions, resistant and susceptible strains, and greater diversity in antibiotic susceptibility profiles would bridge the gap between academic research and clinical implementation. Raman spectroscopy combined with technologies such as hollow-core optical fiber or microscopy would enable the analysis of a single pathogen without time-consuming culturing and complex laboratorial analysis (Neugebauer et al., 2015). The Raman spectral dataset of clinical strains would promote the clinical use of a portable device for field tests. Non-destructive, culture-independent, label-free, and rapid identification of pathogenic microorganisms and the detection of antibiotic susceptibility in patient samples in a single step would be a revolution, improving patient outcomes.

## Data availability statement

The raw data supporting the conclusions of this article will be made available by the authors, without undue reservation.

## Author contributions

WL, HL, and YF designed research. WL analyzed data. HL performed the experiments. HQ and WL discussed the results. JF provided the strains. WL and YF wrote the paper. All authors contributed to the article and approved the submitted version.

## Funding

This research was supported by National Key R&D Program of China (2021YFC2301000 and 2019YFA0905500), the National Natural Science Foundation of China Grant (52091541 and 31970553), Senior User Project of RV KEXUE (KEXUE2019GZ05).

## Conflict of interest

The authors declare that the research was conducted in the absence of any commercial or financial relationships that could be construed as a potential conflict of interest.

## Publisher’s note

All claims expressed in this article are solely those of the authors and do not necessarily represent those of their affiliated organizations, or those of the publisher, the editors and the reviewers. Any product that may be evaluated in this article, or claim that may be made by its manufacturer, is not guaranteed or endorsed by the publisher.

## References

[ref1] Abadi T. B. Rizvanov A. A. Haertlé T. Blatt N. L. (2019). World Health Organization report: current crisis of antibiotic resistance. BioNanoScience 9, 778–788. doi: 10.1007/s12668-019-00658-4

[ref2] Acharya B. Acharya A. Gautam S. Ghimire S. P. Mishra G. Parajuli N.. (2020). Advances in diagnosis of tuberculosis: an update into molecular diagnosis of mycobacterium tuberculosis. Mol. Biol. Rep. 47, 4065–4075. doi: 10.1007/s11033-020-05413-7, PMID: 32248381

[ref3] Andersson D. I. Hughes D. (2014). Microbiological effects of sublethal levels of antibiotics. Nat. Rev. Microbiol. 12, 465–478. doi: 10.1038/nrmicro3270 24861036

[ref4] Antunes L. C. S. Visca P. Towner K. J. (2014). Acinetobacter baumannii: evolution of a global pathogen. Pathogens and Disease 71, 292–301. doi: 10.1111/2049-632X.12125 24376225

[ref5] Aslam B. Wang W. Arshad M. I. Khurshid M. Muzammil S. Rasool M. H.. (2018). Antibiotic resistance: a rundown of a global crisis. Infection and Drug Resistance 11, 1645–1658. doi: 10.2147/IDR.S173867, PMID: 30349322 PMC6188119

[ref6] Ben Y. Fu C. Hu M. Liu L. Wong M. H. Zheng C. (2019). Human health risk assessment of antibiotic resistance associated with antibiotic residues in the environment: a review. Environ. Res. 169, 483–493. doi: 10.1016/j.envres.2018.11.040, PMID: 30530088

[ref7] Biau G. Scornet E. (2016). A random forest guided tour. TEST 25, 197–227. doi: 10.1007/s11749-016-0481-7

[ref8] Butler D. A. Biagi M. Tan X. Qasmieh S. Bulman Z. P. Wenzler E. (2019). Multidrug resistant acinetobacter baumannii: resistance by any other name would still be hard to treat. Curr. Infect. Dis. Rep. 21:46. doi: 10.1007/s11908-019-0706-5, PMID: 31734740

[ref9] Cheng W. Liu M. Liu H. Lin S. (2005). Micro-Raman spectroscopy used to identify and grade human skin pilomatrixoma. Microsc. Res. Tech. 68, 75–79. doi: 10.1002/jemt.20229, PMID: 16228983

[ref10] Choi J. S. Ilin Y. Kraft M. L. Harley B. A. C. (2018). Tracing hematopoietic progenitor cell neutrophilic differentiation via Raman spectroscopy. Bioconjug. Chem. 29, 3121–3128. doi: 10.1021/acs.bioconjchem.8b00459, PMID: 30148625 PMC6346746

[ref11] Cohen J. (1960). A coefficient of agreement for nominal scales. Educ. Psychol. Meas. 20, 37–46. doi: 10.1177/001316446002000104

[ref12] Cui D. Kong L. Wang Y. Zhu Y. Zhang C. (2022). In situ identification of environmental microorganisms with Raman spectroscopy. Environmental Science and Ecotechnology 11:100187. doi: 10.1016/j.ese.2022.100187, PMID: 36158754 PMC9488013

[ref13] Cui F. Yue Y. Zhang Y. Zhang Z. Zhou H. S. (2020). Advancing biosensors with machine learning. ACS Sensors 5, 3346–3364. doi: 10.1021/acssensors.0c01424 33185417

[ref14] Dadgostar P. (2019). Antimicrobial resistance: implications and costs. Infection and Drug Resistance 12, 3903–3910. doi: 10.2147/IDR.S234610, PMID: 31908502 PMC6929930

[ref15] Du J. Su Y. Qian C. Yuan D. Miao K. Lee D.. (2020). Raman-guided subcellular pharmaco-metabolomics for metastatic melanoma cells. Nat. Commun. 11:4830. doi: 10.1038/s41467-020-18376-x, PMID: 32973134 PMC7518429

[ref16] Dukor R. K. (2001). “Vibrational spectroscopy in the detection of cancer” in Handbook of vibrational spectroscopy. ed. Griffiths J. M. C. A. P. R. (Hoboken, New Jersey, US: John Wiley & Sons), 3335–3361.

[ref17] Eliopoulos G. M. Maragakis L. L. Perl T. M. (2008). *Acinetobacter baumannii*: epidemiology, antimicrobial resistance, and treatment options. Clin. Infect. Dis. 46, 1254–1263. doi: 10.1086/529198 18444865

[ref18] Faoláin E. Hunter M. B. Byrne J. M. Kelehan P. Mcnamara M. Byrne H. J.. (2005). A study examining the effects of tissue processing on human tissue sections using vibrational spectroscopy. Vib. Spectrosc. 38, 121–127. doi: 10.1016/j.vibspec.2005.02.013

[ref19] Franco-Duarte R. Černáková L. Kadam S. Kaushik S. Salehi B. Bevilacqua A.. (2019). Advances in chemical and biological methods to identify microorganisms—from past to present. Microorganisms 7:130. doi: 10.3390/microorganisms7050130, PMID: 31086084 PMC6560418

[ref20] García S. Fernández A. Luengo J. Herrera F. (2009). A study of statistical techniques and performance measures for genetics-based machine learning: accuracy and interpretability. Soft. Comput. 13, 959–977. doi: 10.1007/s00500-008-0392-y

[ref21] Germond A. Ichimura T. Horinouchi T. Fujita H. Furusawa C. Watanabe T. M. (2018). Raman spectral signature reflects transcriptomic features of antibiotic resistance in *Escherichia coli*. Communications Biology 1:85. doi: 10.1038/s42003-018-0093-8, PMID: 30271966 PMC6123714

[ref22] Gieroba B. Krysa M. Wojtowicz K. Wiater A. Pleszczyńska M. Tomczyk M.. (2020). The FT-IR and Raman spectroscopies as tools for biofilm characterization created by cariogenic streptococci. Int. J. Mol. Sci. 21:3811. doi: 10.3390/ijms21113811, PMID: 32471277 PMC7313032

[ref23] Hanlon E. B. Manoharan R. Koo T. W. Shafer K. E. Motz J. T. Fitzmaurice M.. (2000). Prospects for *in vivo* Raman spectroscopy. Phys. Med. Biol. 45, R1–R59. doi: 10.1088/0031-9155/45/2/201 10701500

[ref24] He Y. Huang S. Zhang P. Ji Y. Xu J. (2021). Intra-ramanome correlation analysis unveils metabolite conversion network from an isogenic population of cells. MBio 12, e01470–e01421. doi: 10.1128/mBio.01470-21 34465024 PMC8406334

[ref25] He Y. Wang X. Ma B. Xu J. (2019). Ramanome technology platform for label-free screening and sorting of microbial cell factories at single-cell resolution. Biotechnol. Adv. 37:107388. doi: 10.1016/j.biotechadv.2019.04.010, PMID: 31152870

[ref26] Ho C.-S. Jean N. Hogan C. A. Blackmon L. Jeffrey S. S. Holodniy M.. (2019). Rapid identification of pathogenic bacteria using Raman spectroscopy and deep learning. Nat. Commun. 10:4927. doi: 10.1038/s41467-019-12898-9, PMID: 31666527 PMC6960993

[ref27] Jamrozik E. Selgelid M. J. (2020). “Drug-resistant Infection: Causes, consequences, and responses” in Ethics and drug resistance: Collective responsibility for global public health. eds. Jamrozik E. Selgelid M. (Cham: Springer International Publishing), 3–18.

[ref28] Jinshu H. U. Wei L. I. U. Rufu J. I. A. Yuqin S. Chao W. Na T.. (2022). Analysis on the clinical distribution and genomic epidemiological characteristics of carbapenem-resistant *Acinetobacter baumannii*. Microbiology China 49, 270–282.

[ref29] Kendall C. Hutchings J. Barr H. Shepherd N. Stone N. (2011). Exploiting the diagnostic potential of biomolecular fingerprinting with vibrational spectroscopy. Faraday Discuss. 149, 279–290. doi: 10.1039/C005379A 21413186

[ref30] Khan S. Ullah R. Shahzad S. Anbreen N. Bilal M. Khan A. (2018). Analysis of tuberculosis disease through Raman spectroscopy and machine learning. Photodiagn. Photodyn. Ther. 24, 286–291. doi: 10.1016/j.pdpdt.2018.10.014, PMID: 30359757

[ref31] Krafft C. Neudert L. Simat T. Salzer R. (2005). Near infrared Raman spectra of human brain lipids. Spectrochim. Acta A Mol. Biomol. Spectrosc. 61, 1529–1535. doi: 10.1016/j.saa.2004.11.017, PMID: 15820887

[ref32] Kuhar N. Sil S. Umapathy S. (2021). Potential of Raman spectroscopic techniques to study proteins. Spectrochim. Acta A Mol. Biomol. Spectrosc. 258:119712. doi: 10.1016/j.saa.2021.119712, PMID: 33965670

[ref33] Lee K. S. Landry Z. Pereira F. C. Wagner M. Berry D. Huang W. E.. (2021). Raman microspectroscopy for microbiology. Nature Reviews Methods Primers 1:80. doi: 10.1038/s43586-021-00075-6

[ref34] Llor C. Bjerrum L. (2014). Antimicrobial resistance: risk associated with antibiotic overuse and initiatives to reduce the problem. Therapeutic Advances in Drug Safety 5, 229–241. doi: 10.1177/2042098614554919, PMID: 25436105 PMC4232501

[ref35] Lorenz B. Wichmann C. Stöckel S. Rösch P. Popp J. (2017). Cultivation-free Raman spectroscopic investigations of bacteria. Trends Microbiol. 25, 413–424. doi: 10.1016/j.tim.2017.01.002, PMID: 28188076

[ref36] Lu W. Chen X. Wang L. Li H. Fu Y. V. (2020). Combination of an artificial intelligence approach and laser tweezers Raman spectroscopy for microbial identification. Anal. Chem. 92, 6288–6296. doi: 10.1021/acs.analchem.9b04946, PMID: 32281780

[ref37] Maquelin K. Kirschner C. Choo-Smith L. P. Van Den Braak N. Endtz H. P. Naumann D.. (2002). Identification of medically relevant microorganisms by vibrational spectroscopy. J. Microbiol. Methods 51, 255–271. doi: 10.1016/S0167-7012(02)00127-6, PMID: 12223286

[ref38] Michael C. A. Dominey-Howes D. Labbate M. (2014). The antimicrobial resistance crisis: causes, consequences, and management. Front. Public Health 2:145. doi: 10.3389/fpubh.2014.00145 25279369 PMC4165128

[ref39] Movasaghi Z. Rehman S. Rehman I. U. (2007). Raman spectroscopy of biological tissues. Appl. Spectrosc. Rev. 42, 493–541. doi: 10.1080/05704920701551530

[ref40] Murray C. J. L. Ikuta K. S. Sharara F. Swetschinski L. Robles Aguilar G. Gray A.. (2022). Global burden of bacterial antimicrobial resistance in 2019: a systematic analysis. Lancet 399, 629–655. doi: 10.1016/S0140-6736(21)02724-0, PMID: 35065702 PMC8841637

[ref41] Neugebauer U. Clement J. H. Bocklitz T. Krafft C. Popp J. (2010). Identification and differentiation of single cells from peripheral blood by Raman spectroscopic imaging. J. Biophotonics 3, 579–587. doi: 10.1002/jbio.201000020, PMID: 20449831

[ref42] Neugebauer U. Rösch P. Popp J. (2015). Raman spectroscopy towards clinical application: drug monitoring and pathogen identification. Int. J. Antimicrob. Agents 46, S35–S39. doi: 10.1016/j.ijantimicag.2015.10.014 26612228

[ref43] Notingher I. Verrier S. Haque S. Polak J. M. Hench L. L. (2003). Spectroscopic study of human lung epithelial cells (A549) in culture: living cells versus dead cells. Biopolymers 72, 230–240. doi: 10.1002/bip.10378, PMID: 12833477

[ref44] O'neill J. (2016). Tackling drug-resistant infections globally: Final report and recommendations. Available at: https://amr-review.org/Publications.html (Accessed May 19, 2016).

[ref45] Park J. Kim M. Shin B. Kang M. Yang J. Lee T. K.. (2021). A novel decoy strategy for polymyxin resistance in Acinetobacter baumannii. elife 10:e66988. doi: 10.7554/eLife.66988, PMID: 34180396 PMC8324293

[ref46] Parmar A. Katariya R. Patel V. (2019). “A review on random forest: an ensemble classifier” in International conference on intelligent data communication technologies and internet of things (ICICI) 2018. eds. Hemanth J. Fernando X. Lafata P. Baig Z. (Cham: Springer International Publishing), 758–763.

[ref47] Ramirez-Mora T. Dávila-Pérez C. Torres-Méndez F. Valle-Bourrouet G. (2019). Raman spectroscopic characterization of endodontic biofilm matrices. J. Spectrosc. 2019, 1–7. doi: 10.1155/2019/1307397

[ref48] Roope L. S. J. Smith R. D. Pouwels K. B. Buchanan J. Abel L. Eibich P.. (2019). The challenge of antimicrobial resistance: what economics can contribute. Science 364:eaau4679. doi: 10.1126/science.aau4679 30948524

[ref49] Senger R. S. Scherr D. (2020). Resolving complex phenotypes with Raman spectroscopy and chemometrics. Curr. Opin. Biotechnol. 66, 277–282. doi: 10.1016/j.copbio.2020.09.007, PMID: 33142112

[ref50] Shaikhina T. Lowe D. Daga S. Briggs D. Higgins R. Khovanova N. (2019). Decision tree and random forest models for outcome prediction in antibody incompatible kidney transplantation. Biomedical Signal Processing and Control 52, 456–462. doi: 10.1016/j.bspc.2017.01.012

[ref51] Strejcek M. Smrhova T. Junkova P. Uhlik O. (2018). Whole-cell MALDI-TOF MS versus 16S rRNA gene analysis for identification and dereplication of recurrent bacterial isolates. Front. Microbiol. 9:1294. doi: 10.3389/fmicb.2018.01294, PMID: 29971049 PMC6018384

[ref52] Syal K. Mo M. Yu H. Iriya R. Jing W. Guodong S.. (2017). Current and emerging techniques for antibiotic susceptibility tests. Theranostics 7, 1795–1805. doi: 10.7150/thno.19217, PMID: 28638468 PMC5479269

[ref53] Töpfer N. Müller M. M. Dahms M. Ramoji A. Popp J. Slevogt H.. (2019). Raman spectroscopy reveals LPS-induced changes of biomolecular composition in monocytic THP-1 cells in a label-free manner. Integr. Biol. 11, 87–98. doi: 10.1093/intbio/zyz009 31083720

[ref54] Vasala A. Hytönen V. P. Laitinen O. H. (2020). Modern tools for rapid diagnostics of antimicrobial resistance. Front. Cell. Infect. Microbiol. 10:308. doi: 10.3389/fcimb.2020.00308, PMID: 32760676 PMC7373752

[ref55] Vázquez-López R. Solano-Gálvez S. G. Juárez Vignon-Whaley J. J. Abello Vaamonde J. A. Padró Alonzo L. A. Rivera Reséndiz A.. (2020). *Acinetobacter baumannii* resistance: a real challenge for clinicians. Antibiotics 9:205. doi: 10.3390/antibiotics9040205, PMID: 32340386 PMC7235888

[ref56] Verma T. Annappa H. Singh S. Umapathy S. Nandi D. (2021). Profiling antibiotic resistance in *Escherichia coli* strains displaying differential antibiotic susceptibilities using Raman spectroscopy. J. Biophotonics 14:e202000231. doi: 10.1002/jbio.202000231 32981183

[ref57] Vieira S. M. Kaymak U. Sousa J. M. C. (2010). "Cohen's kappa coefficient as a performance measure for feature selection", in *International Conference on Fuzzy Systems*, 1–8.

[ref58] Wieser A. Schneider L. Jung J. Schubert S. (2012). MALDI-TOF MS in microbiological diagnostics—identification of microorganisms and beyond (mini review). Appl. Microbiol. Biotechnol. 93, 965–974. doi: 10.1007/s00253-011-3783-4, PMID: 22198716

[ref59] Woodford N. Ellington M. J. Coelho J. M. Turton J. F. Ward M. E. Brown S.. (2006). Multiplex PCR for genes encoding prevalent OXA carbapenemases in *Acinetobacter spp*. Int. J. Antimicrob. Agents 27, 351–353. doi: 10.1016/j.ijantimicag.2006.01.004, PMID: 16564159

[ref60] Xu Y. Zhong P. Jiang A. Shen X. Li X. Xu Z.. (2020). Raman spectroscopy coupled with chemometrics for food authentication: a review. TrAC Trends Anal. Chem. 131:116017. doi: 10.1016/j.trac.2020.116017

[ref61] Yan S. Qiu J. Guo L. Li D. Xu D. Liu Q. (2021a). Development overview of Raman-activated cell sorting devoted to bacterial detection at single-cell level. Appl. Microbiol. Biotechnol. 105, 1315–1331. doi: 10.1007/s00253-020-11081-1, PMID: 33481066

[ref62] Yan S. Wang S. Qiu J. Li M. Li D. Xu D.. (2021b). Raman spectroscopy combined with machine learning for rapid detection of food-borne pathogens at the single-cell level. Talanta 226:122195. doi: 10.1016/j.talanta.2021.122195, PMID: 33676719

[ref63] Yang K. Li H.-Z. Zhu X. Su J.-Q. Ren B. Zhu Y.-G.. (2019). Rapid antibiotic susceptibility testing of pathogenic bacteria using heavy-water-labeled single-cell Raman spectroscopy in clinical samples. Anal. Chem. 91, 6296–6303. doi: 10.1021/acs.analchem.9b01064, PMID: 30942570

[ref64] Yi X. Song Y. Xu X. Peng D. Wang J. Qie X.. (2021). Development of a fast Raman-assisted antibiotic susceptibility test (FRAST) for the antibiotic resistance analysis of clinical urine and blood samples. Anal. Chem. 93, 5098–5106. doi: 10.1021/acs.analchem.0c04709, PMID: 33728890

[ref65] Zhang M. Hong W. Abutaleb N. S. Li J. Dong P.-T. Zong C.. (2020). Rapid determination of antimicrobial susceptibility by stimulated Raman scattering imaging of D2O metabolic incorporation in a single bacterium. Advanced Science 7:2001452. doi: 10.1002/advs.202001452, PMID: 33042757 PMC7539191

[ref66] Zhou B. Sun L. Fang T. Li H. Zhang R. Ye A. (2022). Rapid and accurate identification of pathogenic bacteria at the single-cell level using laser tweezers Raman spectroscopy and deep learning. J. Biophotonics 15:e202100312. doi: 10.1002/jbio.202100312 35150463

