# Supplementary material for: Identification of pathogens and detection of antibiotic susceptibility at single-cell resolution by Raman spectroscopy combined with machine learning
Source: Front Microbiol. 2023 Jan 4;13:1076965. doi: 10.3389/fmicb.2022.1076965 (PMC9846160; doi:10.3389/fmicb.2022.1076965)
Supplement: Supplementary file 1 [file Data_Sheet_1.pdf]

Supplementary Material

Identification of pathogens and detection of antibiotic susceptibility at single-cell resolution by Raman spectroscopy combined with machine learning

Weilai Lu[1,2†], Haifei Li[1†], Haoning Qiu[1,2], Lu Wang[1,2], Jie Feng[1], Yu Vincent Fu[1,3*]

[1]State Key Laboratory of Microbial Resources, Institute of Microbiology, Chinese Academy of Sciences, Beijing, 100101, China

[2]College of Life Sciences, University of Chinese Academy of Sciences, Beijing, 100049, China

[3]Savaid Medical School, University of Chinese Academy of Sciences, Beijing, 100049, China

*** Correspondence:**
Corresponding Author: Yu Vincent Fu
fuyu@im.ac.cn

Supplementary Figures

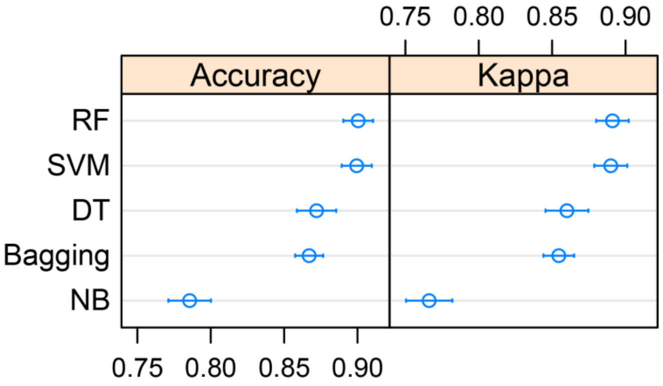

Figure S1. The evaluation of different machine learning methods in 10-fold cross-validation. The blue circles represent the mean value of each model and the error bars represent standard deviation of 10 cross-validation. RF: random forest, SVM: support vector machine, DT: decision tree, NB: naive Bayes.

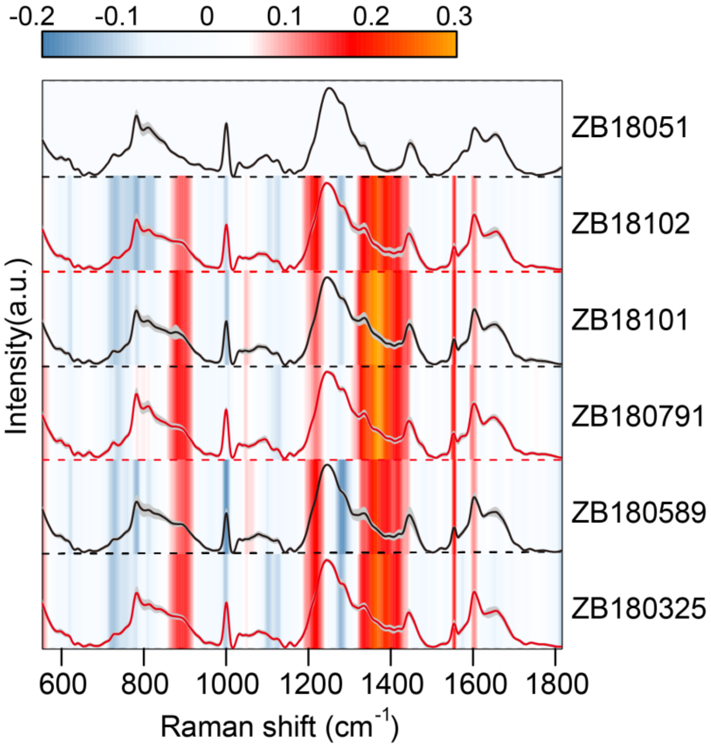

Figure S2. Average Raman spectra of six *A. baumannii* strains. Strain ZB18051 is the antibiotic-sensitive strain, and the strians ZB18102, ZB18101, ZB180791, ZB180589, and ZB180352 are multidrug resistance strains. The average Raman spectra are shown by the solid line, and the standard deviations are represented by the gray shadow. The heatmaps show the sepctral difference between antibiotic-resistant and antibiotic-sensitive bacteria. The difference is indicated by color gradient.

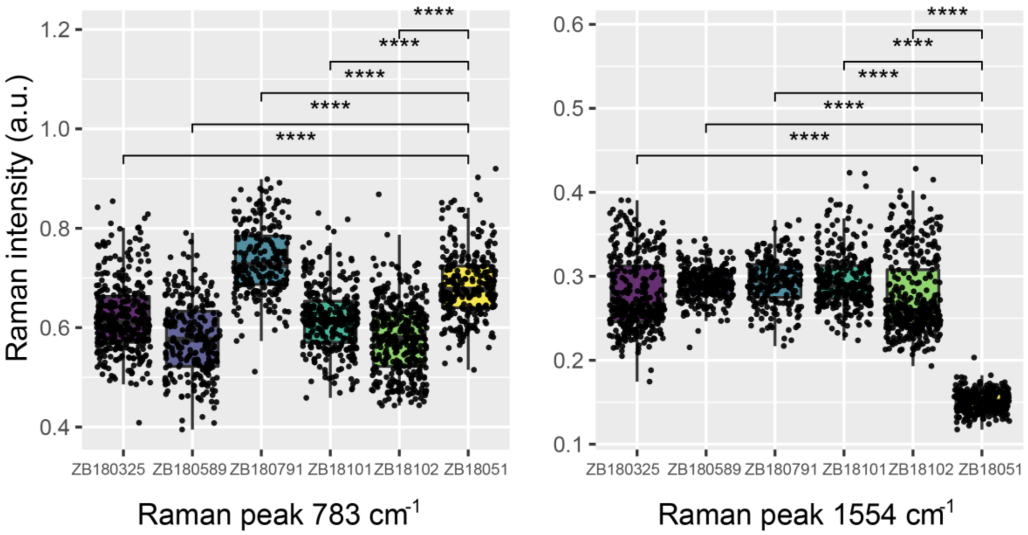

Figure S3. The comparison of Raman intensities between antibiotic-resistant and antibiotic-sensitive *A. baumannii*. Strain ZB18051 is sensitive strain and the others are multidrug resistance strains. Raman intensities among six strains at peaks 783 cm^{-1} and 1554 cm^{-1}. Box plots represent the median and first and third quartiles, with the whiskers representing the minimum and maximum values within 1.5 interquartile ranges from the first and third quartiles. The two sided T-test was applied to compare the statistical significance between antibiotic-resistant and antibiotic-sensitive strains, ****P $\leq$ 0.0001. Each dot represents the Raman intensity of an *A. baumannii* single cell.
