# Supplementary material for: Identification of pathogens and detection of antibiotic susceptibility at single-cell resolution by Raman spectroscopy combined with machine learning
Source: Front Microbiol. 2023 Jan 4;13:1076965. doi: 10.3389/fmicb.2022.1076965 (PMC9846160; doi:10.3389/fmicb.2022.1076965)
Supplement: Supplementary file 2 [file Data_Sheet_2.pdf]

Supplementary Material

Identification of pathogens and detection of antibiotic susceptibility at single-cell resolution by Raman spectroscopy combined with machine learning

Weilai Lu[1,2†], Haifei Li[1†], Haoning Qiu[1,2], Lu Wang[1,2], Jie Feng[1], Yu Vincent Fu[1,3*]

[1]State Key Laboratory of Microbial Resources, Institute of Microbiology, Chinese Academy of Sciences, Beijing, 100101, China

[2]College of Life Sciences, University of Chinese Academy of Sciences, Beijing, 100049, China

[3]Savaid Medical School, University of Chinese Academy of Sciences, Beijing, 100049, China

*** Correspondence:**
Corresponding Author: Yu Vincent Fu
fuyu@im.ac.cn

Supplementary Tables

Table S1. The antimicrobial susceptibility test of *A. baumannii* tested with VITEK 2 system.

Antibiotics	*A. baumannii* ZB180325	*A. baumannii* ZB180589	*A. baumannii* ZB180791	*A. baumannii* ZB18101	*A. baumannii* ZB18102
Imipenem	S	I	S	R	I
Meropenem	I	R	R	R	I
Ampicillin	I	S	R	R	R
Cefoperazone	S	S	I	R	I
Cefepime	I	I	R	R	R
Compound sulfamethoxazole	R	R	R	S	R
Ciprofloxacin	R	R	R	R	R
Ceftazidime	R	R	R	R	R
Ceftriaxone	R	R	R	R	R
Gentamicin	R	R	R	R	R
Levofloxacin	R	R	R	R	R

Note: R: Resistant, I: Intermediate, S: Susceptible

Table S2. Four indicators for evaluating different machine learning models.

Machine learning	Accuracy (%)	Recall (%)	Kappa	F1-score
Random forest	90.73	90.91	0.90	0.91
Support vector machine	90.52	90.79	0.90	0.91
Decision tree	89.27	89.45	0.88	0.89
Bagging	88.13	88.33	0.87	0.88
Naive Bayes	79.90	83.12	0.78	0.86
